# Ultrasensitive Photoelectrochemical Immunoassay Strategy Based on Bi_2_S_3_/Ag_2_S for the Detection of the Inflammation Marker Procalcitonin

**DOI:** 10.3390/bios13030366

**Published:** 2023-03-10

**Authors:** Guanhui Zhao, Yingying Wang, Huixin Wang, Guozhen Bai, Nuo Zhang, Yaoguang Wang, Qin Wei

**Affiliations:** 1 Key Laboratory of Interfacial Reaction & Sensing Analysis in Universities of Shandong, School of Chemistry and Chemical Engineering, University of Jinan, Jinan 250022, China; 2 Shandong Provincial Key Laboratory of Molecular Engineering, School of Chemistry and Chemical Engineering, Qilu University of Technology (Shandong Academy of Sciences), Jinan 250353, China

**Keywords:** photoelectric chemical immunosensor, procalcitonin, semiconductor nanomaterial, Bi_2_S_3_, Ag_2_S

## Abstract

As an inflammatory marker, procalcitonin (PCT) is more representative than other traditional inflammatory markers. In this work, a highly efficient photoelectrochemical (PEC) immunosensor was constructed based on the photoactive material Bi_2_S_3_/Ag_2_S to realize the sensitive detection of PCT. Bi_2_S_3_ was prepared by a hydrothermal method, and Ag_2_S quantum dots were deposited on the ITO/Bi_2_S_3_ surface via in situ reduction. Bi_2_S_3_ is a kind of admirable photoelectric semiconductor nanomaterial on account of its moderate bandgap width and low binding rate of photogenerated electron holes, which can effectively convert light energy into electrical energy. Therefore, based on the energy level matching principle of Bi_2_S_3_ and Ag_2_S, a labeled Bi_2_S_3_/Ag_2_S PEC immunosensor was constructed, and the sensitive detection of PCT was successfully established. The linear detection range of the PEC immunosensor was 0.50 pg∙mL^−1^ to 50 ng∙mL^−1^, and the minimum detection limit was 0.18 pg∙mL^−1^. Compared with the traditional PEC strategy, the proposed PEC immunosensor is simple, convenient, and has good anti-interference, sensitivity, and specificity, which could provide a meaningful theoretical basis and reference value for the clinical detection of PCT.

## 1. Introduction

Procalcitonin (PCT) is an effective marker of blood infection that can be used to guide antibiotic treatment and disease evaluation in patients with respiratory system infections and blood flow infections [1,2]. The PCT levels increase when the patient has inflammation and infection, since the endocrine cells in the lungs and intestinal tissue synthesize and secrete large amounts of PCT [3,4]. The more serious the infection, the higher the PCT content [5,6]. In addition, the level of PCT concentration also plays a guiding role in the diagnosis of sepsis [7,8]. When the concentration of PCT in human serum exceeds 2 ng mL^−1^, it indicates that the blood has an infection that manifests as septicemia [9,10]. In summary, accurate detection of PCT content in serum has important significance for the early prevention and further treatment of inflammation [11,12]. To date, many PCT detection strategies have been reported, including colorimetric immunoassay [13,14], chemiluminescence immunoassay [15], electrochemical immunoassay [16], microfluidic immunoassay, and fluorescence immunoassay [17,18]. Here, we studied the application of PEC immunosensor technology to detect PCT.

PEC immunosensors are a kind of sensing technology that combines photoelectric materials and specific recognition of biomolecules [19]. At present, many biomolecules (e.g., disease markers, gene sequences, specific recognition cells, etc.) have been used as detection targets [20]. The development prospects of immunosensor technology are promising because of its advantages, such as sensitive recognition, high selectivity, simple and easy operation process, and low cost. It is widely used in clinical medicine, food detection, and environmental monitoring [21]. PEC immunosensor is a kind of high-efficiency sensor that combines the advantages of photoelectrochemistry and biochemistry [22]. It has the advantages of miniaturization, simple operation, high sensitivity, and high specificity [23]. Although it has shown many advantages in various fields compared with traditional chemical detection methods, there are still many problems to be solved in the process of PEC technology’s development [24]. Firstly, a more stringent testing environment is required. Secondly, its stability and photoelectric conversion efficiency need to be improved [25]. Therefore, PEC immunosensors not only need bioactive materials with high photoelectric conversion efficiency, a large absorption range, and good stability, but also need to increase the diversity of the detection environment and the tolerance of the sensors [26]. In this work, two kinds of semiconductor materials with excellent photoelectric activity were selected to prepare a photoelectric chemical immunosensor that possessed the advantages of high photoelectric conversion efficiency, high stability, and simple determination [27].

Bi_2_S_3_ belongs to a family of metal chalcogenides in a class of non-toxic semiconductor materials [28], whose importance in photovoltaic and thermoelectric applications is well recognized [29]. Bi_2_S_3_ is an excellent photoelectric material because of its moderate bandgap width and low binding rate of photogenerated electron holes, which can effectively convert light energy into electrical energy [30]. Therefore, Bi_2_S_3_ was selected as the base material of the immunosensor in this work. In addition, Bi_2_S_3_ was sensitized by in situ deposition of Ag_2_S quantum dots via an immersion method. The preparation process was simple, universal, green, and excellent. The Bi_2_S_3_/Ag_2_S modified electrode significantly improved the photocurrent signal intensity of the sensor substrate, laying a good foundation for the construction of the PEC immunosensor. In this work, rod-like Bi_2_S_3_ was synthesized by a hydrothermal method, and Ag_2_S quantum dots were deposited in situ via an immersion strategy. Based on this, Bi_2_S_3_/Ag_2_S was used as the base material of the electrode, and anti-PCT, bovine serum albumin (BSA), and PCT were modified layer by layer; thus, a new and high-efficiency PEC immunosensor was constructed to realize the sensitive detection of PCT, and the limit of detection (LOD) was 0.18 pg mL^−1^. Compared with traditional sensors, the preparation process was simple and had fewer interference factors. The sensitivity, selectivity, and stability were excellent. This work could provide a theoretical basis for the use of semiconductor materials in sensing analysis.

## 2. Materials and Methods

### 2.1. Materials

Anti-PCT and PCT were obtained from Lingchao Biotechnology Co., Ltd. (Shanghai, China). The other materials and apparatus used are discussed in the Supplementary Materials.

### 2.2. Synthesis Procedure of Bi_2_S_3_

The Bi_2_S_3_ was synthesized via a one-step hydrothermal method as reported in the literature [31]. First, 0.2254 g of thioacetamide and 0.7902 g of anhydrous bismuth nitrate were mixed and dissolved in 40 mL of ethanol, which was stirred to completely dissolve the mixture and then transferred to high-pressure Teflon-lined stainless-steel autoclave for 12 h at 180 °C. The product was washed with ultrapure water and ethanol and dried at 70 °C for 12 h in a vacuum-drying oven to obtain Bi_2_S_3_ as a black powder.

### 2.3. Steps of Synthesis of Bi_2_S_3_/Ag_2_S

On the basis of the previous literature, Ag_2_S was deposited in situ via an immersion method. First, the ITO modified with Bi_2_S_3_ was immersed in 0.1 mol L^−1^ AgNO_3_ solution prepared with ethanol for 3 min, and then washed gently with ethanol. After natural drying at room temperature, it was immersed in 0.1 mol L^−1^ Na_2_S prepared by blending methanol and ultrapure water (volume ratio 1:1) for 3 min. Then, it was washed with a mixture of methanol and water (volume ratio 1:1). After drying at room temperature, Ag_2_S was successfully deposited on the surface of the Bi_2_S_3_/ITO electrode.

### 2.4. The Establishment Process of the Proposed PEC Immunosensor

Firstly, the ITO glass electrode was cut to 2.5 × 1.0 cm^2^. Then, the ITO was washed ultrasonically with acetone, ethanol, and ultrapure water solution successively for 30 min. Then, the ITO was dried at 70 °C in the vacuum oven for 2 h. Next, we took 6 μL of Bi_2_S_3_ suspension solution (concentration = 5 mol L^−1^), applied it to the surface of the clean ITO electrode, and allowed it to dry naturally at room temperature until slightly wet. Then, the Bi_2_S_3_/ITO electrode was immersed in 0.1 mol L^−1^ AgNO_3_ solution for 3 min and then gently washed once with ethanol after being taken out with a clip. After it was dried naturally at room temperature, the ITO was immersed in 0.1 mol L^−1^ Na_2_S solution for 3 min. Then, it was taken out with a clip and washed twice with a mixture of methanol and ultrapure water (1:1). Thus, the synthetic deposition of Ag_2_S quantum dots was successful, and the Ag_2_S/Bi_2_S_3_/ITO modified electrode was successfully prepared.

**Scheme 1:**
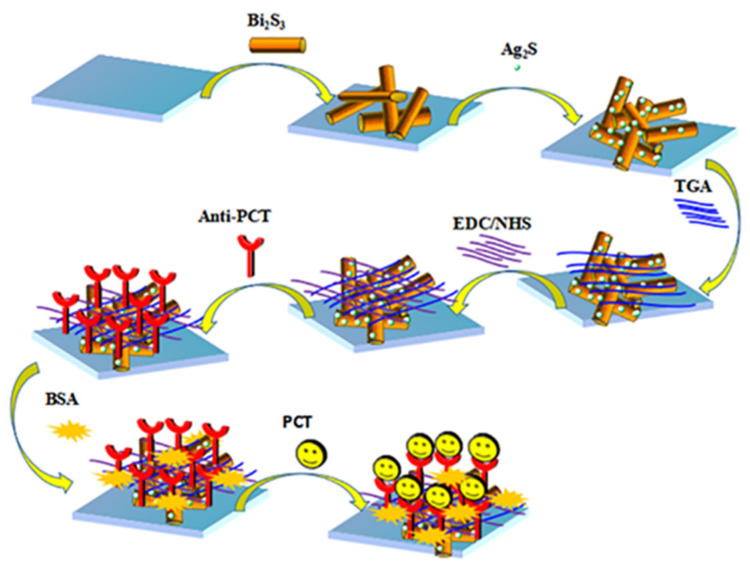
Schematic diagram of the PEC sensor’s construction.

Then, we added 6 μL of 3 mmol L^−1^ TGA solution onto the prepared Ag_2_S/Bi_2_S_3_/ITO surface, and the carboxyl group in TGA and the amino group in the anti-PCT could undergo a condensation reaction and connect with one another. Subsequently, 6 μL of EDC/NHS (1:1) mixture was dropped onto the ITO to connect the anti-PCT with the base material and dried at 4 °C in a refrigerator until the surface was slightly wet. Then, 6 μL of PBS solution containing 0.1% BSA was dropped to block non-specific binding sites on the surface of the Ag_2_S/Bi_2_S_3_/ITO. Finally, we added different concentrations of PCT and dried them at 4 °C in the refrigerator until the surface was slightly wet. It is worth noting that in each step of the electrode modification process, PBS solution was used once to wash away the excess substances not participating in the reaction when the electrode surface was dried to slightly wet. At this point, we had completed the construction of the PCT/BSA/anti-PCT/EDC/NHS/Ag_2_S/Bi_2_S_3_/ITO immunosensor, and it was then stored at 4 °C for standby. The construction diagram of the sensor is shown in Scheme 1.

### 2.5. PEC Analysis of PCT

The photocurrent signal of this PEC sensor was measured with a three-electrode system on the photoelectrochemical workstation. The three-electrode system included a saturated calomel electrode (reference electrode), a counter electrode, and a working electrode. The calomel electrode was easily affected by the concentration and temperature of potassium chloride, so it was necessary to ensure that the potassium chloride solution was saturated. The counter electrode used in this workstation was a platinum counter electrode. As the electronic conductor of the other two electrodes, it did not participate in the electrode reaction. The Bi_2_S_3_/Ag_2_S modified ITO glass electrode was used as the working electrode.

We placed the three-electrode system in PBS (pH = 7.4) solution containing 0.14 mol L^−1^ ascorbic acid (AA), and the light source was an LED lamp. The photocurrent signal of the sensor was detected by chronoamperometry, and the working curve was constructed according to the relationship between the photocurrent signal and the logarithm of the concentration of PCT antigen, so as to achieve the detection of the concentration level of the inflammatory marker PCT.

## 3. Results and Discussion

### 3.1. The Characteristics of Bi_2_S_3_ and Bi_2_S_3_/Ag_2_S

**Figure 1:**
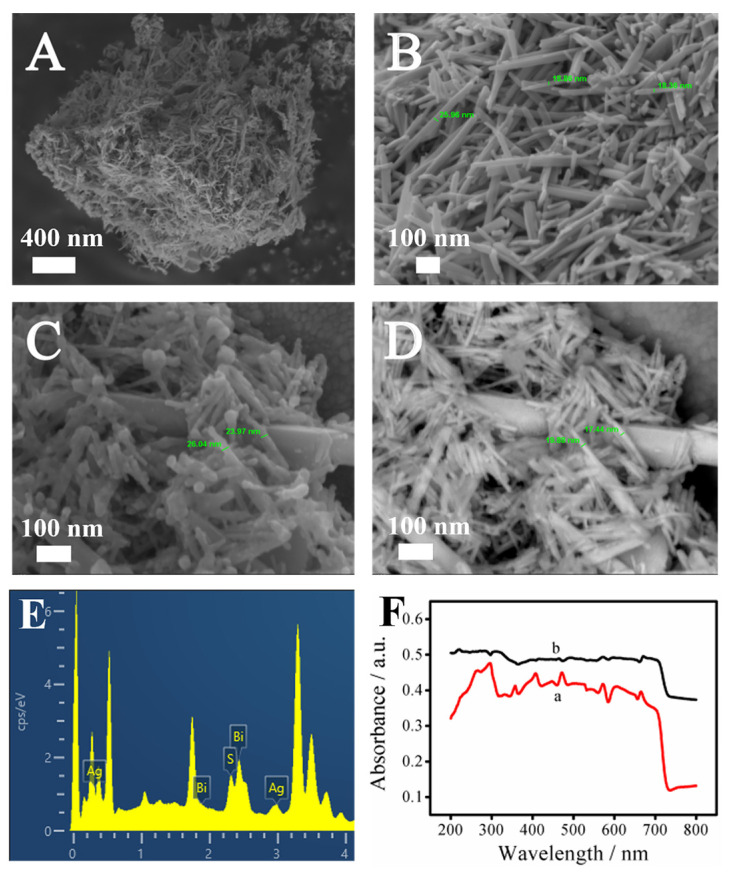
SEM images (**A**,**B**) of Bi_2_S_3_ with different magnification; SEM diagrams (**C**,**D**) of Bi_2_S_3_/Ag_2_S under InLens and BSE detectors, respectively; Energy spectrum analysis (EDS) diagram (**E**) of Bi_2_S_3_/Ag_2_S; Ultraviolet–visible (UV–vis) near-infrared absorption spectra (**F**) of Bi_2_S_3_ (a) and Ag_2_S (b).

Field-emission scanning electron microscopy (SEM) was used to observe the nanoscale morphology and structure of Bi_2_S_3_ and the Bi_2_S_3_/Ag_2_S composites to determine whether the synthesized materials met the requirements and whether Ag_2_S was successfully loaded onto the Bi_2_S_3_ materials, as shown in Figure 1. Figure 1A,B show the SEM images of Bi_2_S_3_ at different magnifications. It can be seen that Bi_2_S_3_ was a block composed of nanorods of different lengths and thicknesses crisscrossed together. Figure S1 shows the X-ray diffraction of Bi_2_S_3_, where it can be seen that, compared with the standard card, the XRD spectrum covered the most of the characteristic peaks of Bi_2_S_3_. Figure 1B shows that Bi_2_S_3_ was actually scattered nanorods with a diameter of 17~26 nm. The cylindrical side of the rod-shaped structure could provide a greater specific surface area and more active sites for the loading of Ag_2_S.

Figure 1C,D show the SEM images of Bi_2_S_3_/Ag_2_S material under two detectors (InLens and BSE, respectively). The BSE detector was mainly used for signal scanning of backscattered electrons. The higher the atomic number, the stronger the signal, and the brighter the image. By comparing the brightness and size in Figure 1C,D, it can be seen that the Ag_2_S quantum dots were uniformly attached to the surface of the Bi_2_S_3_.

At the same time, the elements contained in the material were analyzed with an energy spectrometer, as shown in Figure 1E. It could be seen that the base material contained the elements Bi, S, and Ag, and the Ag was uniformly distributed on the surface of the Bi_2_S_3_, further proving that Bi_2_S_3_ and Ag_2_S were successfully modified on the surface of the ITO electrode. In addition, the EDS mapping images of the Bi_2_S_3_/Ag_2_S composites are shown in Figure S2.

In order to further prove that the Ag_2_S quantum dots were successfully deposited on the Bi_2_S_3_ nanorods, the UV–vis absorption spectra of Bi_2_S_3_ and Bi_2_S_3_/Ag_2_S were measured, as shown in Figure 1F. It could be observed that when the Ag_2_S quantum dots were deposited on the Bi_2_S_3_, the absorbance of Bi_2_S_3_/Ag_2_S (b curve) increased significantly and showed slight redshifts, indicating that Ag_2_S has a certain sensitization effect on Bi_2_S_3_.

**Figure 2:**
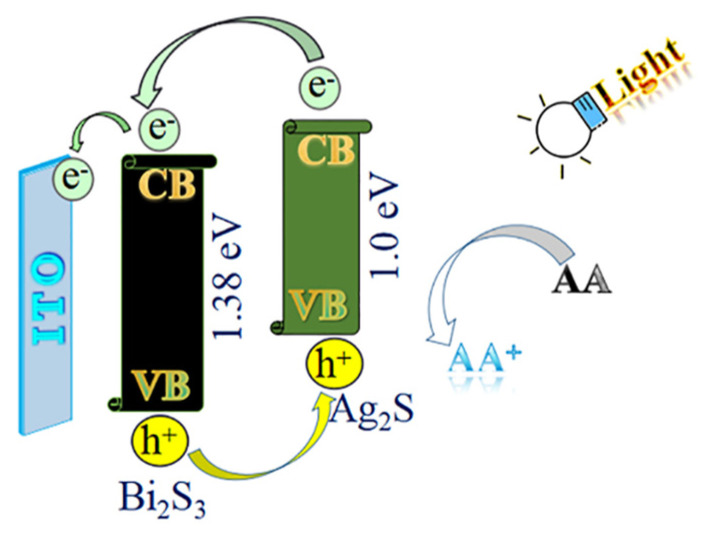
The electron transfer mechanism of the PEC immunosensor.

The satisfying photoelectric performance of the substrate material was dependent on its electron transfer mechanism. As shown in Figure 2, Bi_2_S_3_ has a bandgap energy of about 1.38 eV. Under the illumination of the light source, the absorbed light energy of electrons in the valence band of Bi_2_S_3_ transitioned to the conduction band. Thus, the holes were generated in the valence band and the electrons were increased in the conduction band. The electron holes moved and formed the current circuit, which successfully converted light energy into electric energy. AA, as an electron donor, could provide electrons and neutralize excessive photogenerated holes in the valence band, inhibiting the recombination of electron–hole pairs, enhancing the continuity of the current circuit, and improving the output efficiency of the photocurrent. Thus, the photoelectric activity of Bi_2_S_3_ could be improved. In addition, the generation principle of the electron–hole pairs was the same as described above when the light source was irradiated on the Ag_2_S quantum dots. The electrons in the conduction band of the Ag_2_S quantum dots were transferred to the conduction band of the Bi_2_S_3_ and then transferred to the electrode surface, forming an electron gradient flow. The energy band matching of the Bi_2_S_3_ and the Ag_2_S quantum dots effectively prevented the electron–hole recombination of Ag_2_S. Therefore, the electrons on the Ag_2_S could be efficiently transferred to the electrode surface to increase the photocurrent.

### 3.2. The Performance Characterization of the Proposed PEC Immunosensor

**Figure 3:**
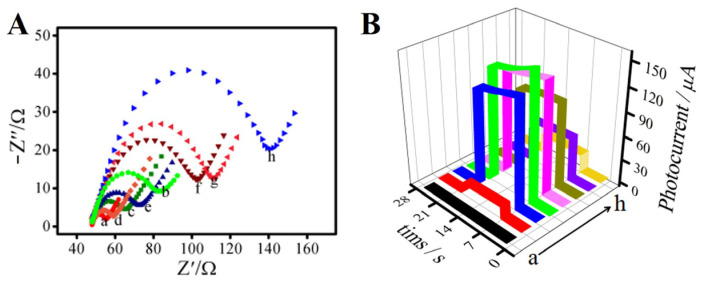
The electrochemical impedance spectroscopy (EIS) (**A**) and the chronoamperogram (**B**) of the photoelectrochemical immunosensor: (a) ITO; (b) Bi_2_S_3_/ITO; (c) Ag_2_S/Bi_2_S_3_/ITO; (d) TGA/Ag_2_S/Bi_2_S_3_/ITO; (e) EDC/NHS/TGA/Ag_2_S/Bi_2_S_3_/ITO; (f) anti-PCT/EDC/NHS/TGA/Ag_2_S/Bi_2_S_3_/ITO; (g) BSA/anti-PCT/EDC/NHS/TGA/Ag_2_S/Bi_2_S_3_/ITO; (h) PCT/BSA/anti-PCT/EDC/NHS/TGA/Ag_2_S/Bi_2_S_3_/ITO.

Figure 3 shows the electrochemical impedance spectroscopy (EIS) characteristics of the immunosensor in PBS (pH = 7.4) solution containing 2.5 mmol L^−1^ [Fe(CN)_6_]^3−/4−^ and 5 mmol L^−1^ KNO_3_. As shown in Figure 3A, with the layer-by-layer modification of the electrode surface, the semicircle’s diameter changed accordingly. Curve (a) shows the bare ITO glass electrode, and its semicircle diameter is very small, which indicates that its electron transfer resistance was very low. When the Bi_2_S_3_ (curve b) nanorod was modified to the electrode surface, it could be seen that it had a certain impedance because Bi_2_S_3_ is a nano-semiconductor material. However, the impedance value decreased instead (curve c) when Ag_2_S was deposited on the Bi_2_S_3_/ITO surface. Curves (d) and (e) show the impedance changes after successful modification of the coupling agents TGA and EDC/NHS, respectively. The impedance value decreased after the addition of TGA (curve d), which may have been due to the acidic nature of TGA. This could make the base liquid around the tested electrode present a strong electrolyte solution, resulting in an increase in the action of free electron transfer. After dropping with EDC/NHS (curve e), the diameter of the semicircle increased. We hypothesized that the EDC/NHS activated the carboxyl groups and reduced the acidity of TGA, so the free electron transfer decreased and the impedance value increased. The impedance value increased significantly (curve f) when the antibody, BSA, and PCT were successfully deposited in sequence, because they are biological protein molecules. As shown in Figure 3B, we studied the timing currents of the electrodes at different modification steps. It can be seen from curve (a) that the timing current of the bare ITO electrode was almost zero. The photocurrent signal increased significantly to 16 μA when the electrode surface was modified with Bi_2_S_3_ (curve b), because Bi_2_S_3_ is an efficient photoelectric semiconductor material. After depositing Ag_2_S on the surface of Bi_2_S_3_ (curve c), the current signal increased significantly to about 130 µA because of the energy band matching principle of Bi_2_S_3_ and Ag_2_S. After the coupling agents TGA (curve d) and EDC/NHS (curve e) were deposited onto the Ag_2_S/Bi_2_S_3_/ITO surface in sequence, the photocurrent change was inversely proportional to the EIS. When the antibody (curve f), BSA (curve g), and PCT were continuously deposited on the Ag_2_S/Bi_2_S_3_/ITO surface, the current signal decreased successively, because all of these substances are biological protein molecules that could block the electronic transmission.

### 3.3. Optimal Conditions for Analysis 

**Figure 4:**
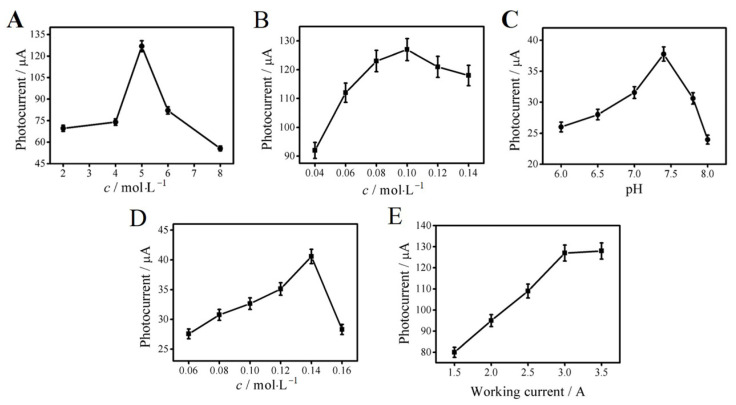
Optimal concentrations of Bi_2_S_3_ (**A**), AgNO_3_ (**B**), and AA (**D**); pH (**C**); photocurrent intensity (**E**). Error bar = SD (*n* = 5).

The different concentrations of Bi_2_S_3_ suspensions were prepared to drop onto the electrode. The photocurrent detection results are shown in Figure 4A. It can be seen that excessive concentrations of Bi_2_S_3_ suspension would make the resistance greater than the conductivity. Therefore, 5 mg mL^−1^ of Bi_2_S_3_ suspension was selected. As shown in Figure 4B, the thick material layer formed by the surplus Ag_2_S nanoparticles could reduce the conductivity and electron transfer rate and increase the photogenerated electron–hole binding rate. Therefore, 0.1 mol L^−1^ of AgNO_3_ solution was finally selected as the best concentration. Figure 4C shows that the photocurrent value reached its maximum when the pH was 7.4. Thus, PBS buffer with pH 7.4 was selected in this experiment. It can be seen from Figure 4D that the photocurrent response was the strongest when the concentration of AA was 0.14 mol L^−1^. The decrease in the photocurrent signal at higher concentrations of AA could be because of the quenching absorption of the electrolyte solution, which reduced the formation efficiency and light intensity of the excited electron–hole center. As shown in Figure 4E, the photocurrent signal intensity reached about 127 µA and then remained essentially unchanged when the working current was 3.0 A. In order to avoid the impact of strong light on photoelectric materials and biomolecules, 3.0 A was selected as the best working current.

### 3.4. PCT Detection

**Figure 5:**
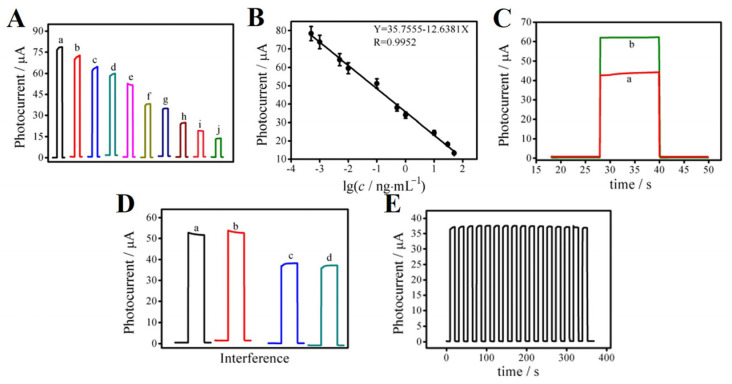
Photocurrent response curves (**A**) and the linearity correction curve (**B**) of the PEC immunosensor at different PCT concentrations: (a) 0.0005, (b) 0.001, (c) 0.005, (d) 0.01, (e) 0.1, (f) 0.5, (g) 1.0, (h) 10, (i) 30, (j) 50 ng mL^−1^. Photocurrent curve of unknown PCT concentration (**C**): the mixture of PCT with concentrations of (a) 0.1 ng mL^−1^ and 0.5 ng mL^−1^, (b) 0.01 ng mL^−1^ and 0.005 ng mL^−1^. The specificity of the constructed immunosensor (**D**): (a) blank, (b) blank + 100 ng mL^−1^ BNP, (c) 0.50 ng mL^−1^ PCT, (d) 0.50 ng mL^−1^ + 100 ng mL^−1^ BNP. Stability evaluation of the photoelectric chemical immunosensor under multiple on/off irradiation cycles for 400 s (**E**). Error bars = SD (*n* = 5).

Under the optimal experimental conditions, the photocurrent response curves were as shown in Figure 5A. Because PCT and PCT-antibody are not conductive, they could hinder the transfer of electrons between the electrode surface and the substrate solution. Thus, the response current continued to decrease with the increase in the PCT concentration and presented a good linear relationship, as shown in Figure 5B. The linear equation was *I* (μA) = 35.7555–12.6381 log *c*; linear correlation coefficient *R*^2^ = 0.9952. The linear detection range of PCT for this proposed immunosensor was 0.5 pg mL^−1^~50 ng mL^−1^, and the detection limit was as low as 0.18 pg mL^−1^. (S/N = 3). In order to verify the accuracy of the constructed PEC immunosensor, four samples with known PCT concentrations were mixed and dropped onto the constructed immunosensor. In Figure 5C, curve (a) shows the photocurrent curve of the mixture of two known PCT concentrations (0.1 ng mL^−1^ and 0.5 ng mL^−1^). According to the linear fitting formula, the theoretical photocurrent intensity was 42.0 μA. The actual photocurrent intensity was 44.65 μA. Curve (b) shows the photocurrent curve of the mixture of two known PCT concentrations (0.01 ng mL^−1^ and 0.005 ng mL^−1^). According to the linear fitting formula, the theoretical photocurrent intensity was 62.0 μA. The actual photocurrent intensity was 63.75 μA, and the relative deviation was 2.8% and 5.9%, respectively, showing that the accuracy of this PEC immunosensor was high and met the experimental needs. Comparing this sensor with other sensors (Table S1), it can be concluded that this PEC sensor has a wide linear range and a low detection limit, indicating that it has relatively excellent performance in detecting PCT, so this work is valuable.

### 3.5. Specificity, Stability, and Application of the PEC Immunosensor

In order to study the specificity of the PEC immunosensor, we selected the interfering substance B-type natriuretic peptide (BNP). In Figure 5D, curve (a) shows the photoelectric signal response of the blank sample. Curve (b) shows the photoelectric signal response of a mixture of BNP (100 ng mL^−1^) and blank. Curve (c) shows the photoelectric signal response of the PCT sample (0.50 ng mL^−1^). Curve (d) shows the photoelectric signal response of the sample containing PCT (0.50 ng mL^−1^) and BNP (100 ng mL^−1^). The prepared sensor was determined according to the experimental method. Adding BNP had no obvious effect on the current signal of the PEC immunosensor, indicating that it had good specificity. To test the stability of the PEC immunosensor, the light was repeatedly switched on and off 18 times in 400 s to detect the photocurrent. The experimental results are shown in Figure 5E. The relative standard deviation (RSD) was 0.047, and the coefficient of variation (CV) was 4.7% relative to the stability experiment. The difference between the timing current after 18 on/off cycles and the initial timing current was very small, indicating that the PEC immunosensor has good operational stability. In addition, seven sensors were stored in a refrigerator at 4 °C for five days, and the results showed that the current value of the immunosensor changed by less than 4%, indicating that the sensor has good storage stability. To study the potential application significance of the proposed PEC immunosensor for PCT determination, the standard addition recovery tests were implemented. Before that, we conducted pre-treatment for serum samples, including low-speed centrifugation at 4 °C, removing sediments, and using the supernatant for testing. As shown in Table S2, the recoveries were in the range of 86~101%, and the RSD was in the range of 3.1~4.1%, indicating great reference significance for clinical PCT detection.

## 4. Conclusions

In this work, since the complex of Bi_2_S_3_/Ag_2_S has excellent photoelectric performance, it was used as the base material to construct the PEC immunosensor. In addition, anti-PCT, BSA, and PCT were successively deposited on the Bi_2_S_3_/Ag_2_S/ITO surface. The novel unmarked PEC immunosensor was constructed successfully and achieved the highly sensitive detection of PCT in human serum. Under the optimal experimental conditions, the linear range of the PEC immunosensor for PCT detection was 0.5 pg mL^−1^~50.0 ng mL^−1^, and the detection limit was as low as 0.18 pg mL^−1^. Moreover, the PEC immunosensor showed admirable stability, selectivity, and reproducibility. This could provide a theoretical basis and reference value for the clinical application of semiconductor materials in the field of disease marker detection.

## Data Availability

The data presented in this study are available upon request from the corresponding authors.

## Supplementary Materials

The following supporting information [8,32,33,34,35,36] can be downloaded at https://www.mdpi.com/article/10.3390/bios13030366/s1: Materials; Apparatus; Figure S1: The XRD of Bi_2_S_3_; Figure S2: The EDS mapping images of Bi_2_S_3_/Ag_2_S composites; Table S1: Comparison of the performance of the proposed and reference methods for PCT detection; Table S2: The results of the PCT determination in human serum samples.

## References

[1] Wussler D., Kozhuharov N., Oliveira M.T., Bossa A., Sabti Z., Nowak A., Murray K., de Lavallaz J.F., Badertscher P., Twerenbold R. (2019). Clinical utility of procalcitonin in the diagnosis of pneumonia. Clin. Chem..

[2] Brabenec L., Hellenthal K., Müller M., Kintrup S., Zurek-Leffers F., Kardell M., Otto M., Wagner N.-M. (2022). Procalcitonin mediates vascular dysfunction in obesity. Life Sci..

[3] Wolfisberg S., Gregoriano C., Schuetz P. (2022). Procalcitonin for individualizing antibiotic treatment: An update with a focus on COVID-19. Crit. Rev. Clin. Lab. Sci..

[4] Battaglia F., Baldoneschi V., Meucci V., Intorre L., Minunni M., Scarano S. (2021). Detection of canine and equine procalcitonin for sepsis diagnosis in veterinary clinic by the development of novel MIP-based SPR biosensors. Talanta.

[5] Jia Y., Yang L., Xue J., Zhang N., Fan D., Ma H., Ren X., Hu L., Wei Q. (2019). Bioactivity-protected electrochemiluminescence biosensor using gold nanoclusters as the low-potential luminophor and Cu_2_S snowflake as co-reaction accelerator for procalcitonin analysis. ACS Sens..

[6] Zhu K., Chen J., Hu J., Xiong S., Zeng L., Huang X., Xiong Y. (2022). Low-sample-consumption and ultrasensitive detection of procalcitonin by boronate affinity recognition-enhanced dynamic light scattering biosensor. Biosens. Bioelectron..

[7] Ge X., Zhang J., Feng Y., Wang A., Mei L., Feng J.J. (2022). Label-free electrochemical biosensor for determination of procalcitonin based on graphene-wrapped Co nanoparticles encapsulated in carbon nanobrushes coupled with AuPtCu nanodendrites. Microchim. Acta.

[8] Miao J., Du K., Li X., Xu X., Dong X., Fang J., Cao W., Wei Q. (2021). Ratiometric electrochemical immunosensor for the detection of procalcitonin based on the ratios of SiO_2_-Fc-COOH–Au and UiO-66-TB complexes. Biosens. Bioelectron..

[9] Zhao L., Song X., Ren X., Wang H., Fan D., Wu D., Wei Q. (2021). Ultrasensitive near-infrared electrochemiluminescence biosensor derived from Eu-MOF with antenna effect and high efficiency catalysis of specific CoS_2_ hollow triple shelled nanoboxes for procalcitonin. Biosens. Bioelectron..

[10] Song X., Zhao L., Zhang N., Liu L., Ren X., Ma H., Kuang X., Li Y., Luo C., Wei Q. (2023). Ultrasensitive electrochemiluminescence biosensor with silver nanoclusters as a novel signal probe and α-Fe_2_O_3_-Pt as an efficient co-reaction accelerator for procalcitonin immunoassay. Anal. Chem..

[11] Zhang N., Feng J., Zhao G., Duan X., Wang Y., Zhang D., Wei Q. (2021). Ultrasensitive photochemical immunosensor based on flowerlike SnO_2_/BiOI/Ag_2_S composites for detection of procalcitonin. Biosensors.

[12] Gupta Y., Pandey C.M., Ghrera A.S. (2023). Development of conducting cellulose paper for electrochemical sensing of procalcitonin. Microchim. Acta.

[13] Li Y., Liu L., Wang Y., Ren R., Fan D., Wu D., Du Y., Xu K., Ren X., Wei Q. (2020). Enzyme-free colorimetric immunoassay for procalcitonin based on MgFe_2_O_4_ sacrificial probe with the Prussian blue production. Sens. Actuators B-Chem..

[14] Zhang Y., Si X., Zhang M., Yang X., Yuan H., Wang X., Zhang Y., Wang H. (2019). Rapid colorimetric determination of procalcitonin using magnetic separation and enzymatic catalysis. Anal. Lett..

[15] Huang E., Huang D., Wang Y., Cai D., Luo Y., Zhong Z., Liu D. (2022). Active droplet-array microfluidics-based chemiluminescence immunoassay for point-of-care detection of procalcitonin. Biosens. Bioelectron..

[16] Tanak A.S., Jagannath B., Tamrakar Y., Muthukumar S., Prasad S. (2019). Non-faradaic electrochemical impedimetric profiling of procalcitonin and C-reactive protein as a dual marker biosensor for early sepsis detection. Anal. Chim. Acta X.

[17] Molinero-Fernández Á., López M., Escarpa A. (2020). An on-chip microfluidic-based electrochemical magneto-immunoassay for the determination of procalcitonin in plasma obtained from sepsis diagnosed preterm neonates. Analyst.

[18] Molinero-Ferna A.G., Moreno-Guzman M., Arruza L., Lo M.A.N., Escarpa A. (2020). Polymer-based micromotor fluorescence immunoassay for on-the-move sensitive procalcitonin determination in very low birth weight infants’ plasma. ACS Sens..

[19] Cao Y., Wang L., Wang C., Hu X., Liu Y., Wang G. (2019). Sensitive detection of glyphosate based on a Cu-BTC MOF/g-C_3_N4 nanosheet photoelectrochemical sensor. Electrochim. Acta.

[20] Afzal A., Iqbal M., Dastgeer G., Nazir G., Mumtaz S., Usman M., Eom J. (2020). WS_2_/GeSe/WS_2_ bipolar transistor-based chemical sensor with fast response and recovery times. ACS Appl. Mater. Inter..

[21] Aydin M., Aydin E., Sezgintürk M. (2021). Advances in immunosensor technology. Adv. Clin. Chem..

[22] Zhang J., Xue X., Du Y., Zhao J., Ma H., Ren X., Wei Q., Ju H. (2022). Antigen-down PEC immunosensor for Cyfra21-1 detection based on photocurrent polarity switching strategy. Anal. Chem..

[23] Liu K., Deng H., Wang Y., Cheng S., Xiong X., Li C. (2020). A sandwich-type photoelectrochemical immunosensor based on ReS_2_ nanosheets for high-performance determination of carcinoembryonic antigen. Sensor. Actuators B-Chem..

[24] Li H., Mu Y., Yan J., Cui D., Ou W., Wan Y., Liu S. (2015). Label-free photoelectrochemical immunosensor for neutrophil gelatinase-associated lipocalin based on the use of nanobodies. Anal. Chem..

[25] Lu Y., Wang H., Shi X., Ding C., Fan G. (2022). Photoanode-supported cathodic immunosensor for sensitive and specific detection of human chorionic gonadotropin. Anal. Chim. Acta.

[26] Wang J., Guo Q., Li Q., Zheng L., Yang X., Wang X., Nie G. (2022). A “signal-off” type photoelectrochemical immunosensor for detecting carcinoembryonic antigen based on TiO_2_ NRs/BiOI heterojunction and SiO_2_/PDA-Au inhibitor. Microchem. J..

[27] Zhang L., Ran J., Qiao S., Jaroniec M. (2019). Characterization of semiconductor photocatalysts. Chem. Soc. Rev..

[28] Jia Y., Liu P., Wang Q., Wu Y., Cao D., Qiao Q. (2021). Construction of Bi_2_S_3_-BiOBr nanosheets on TiO_2_ NTA as the effective photocatalysts: Pollutant removal, photoelectric conversion and hydrogen generation. J. Colloid Interf. Sci..

[29] Wang B., Cao J., Dong Y., Liu F., Fu X., Ren S., Ma S., Liu Y. (2018). An in situ electron donor consumption strategy for photoelectrochemical biosensing of proteins based on ternary Bi_2_S_3_/Ag_2_S/TiO_2_ NT arrays. Chem. Commun..

[30] Xiao W., Wang L., Wei X., Li J. (2022). Chitosan-based molecularly imprinted photoelectric sensor with ZnO/Bi_2_O_3_/Bi_2_S_3_ sensing layer for thiamethoxam determination. Microchim. Acta.

[31] Shi H., Zhao Y., Fan J., Tang Z. (2019). Construction of novel Z-scheme flower-like Bi_2_S_3_/SnIn_4_S_8_ heterojunctions with enhanced visible light photodegradation and bactericidal activity. Appl. Surf. Sci..

[32] Li Y., Liu L., Liu X., Ren Y., Xu K., Zhang N., Sun X., Yang X., Ren X., Wei Q. (2020). A dual-mode PCT electrochem-ical immunosensor with CuCo2S4 bimetallic sulfides as enhancer. Biosens. Bioelectron..

[33] Chiang C., Huang T., Wang C., Huang C., Tsai T., Yu S., Chen Y., Hong S., Hsu C., Chang T. (2020). Fiber optic nanogold-linked immunosorbent assay for rapid detection of procalcitonin at femtomolar concentration level. Biosens. Bioelectron..

[34] Zhou Y., Shao X., Han Y., Zhang H. (2018). Detection of procalcitonin (PCT) using the double antibody sandwich method based on fluorescence resonance energy transfer between upconversion nanoparticles and quantum dots. Anal. Methods.

[35] Wang X., Ma L., Hu C., Liu T., Sun S., Liu X., Guan M. (2021). Simultaneous quantitative detection of IL-6 and PCT using SERS magnetic immunoassay with sandwich structure. Nanotechnology.

[36] Xu X., Lei X., Ye L., Song S., Liu L., Xu L., Xu C., Hua K. (2022). Gold-based paper sensor for sensitive detection of procalcitonin in clinical samples. Chinese J. Anal. Chem..

