# Supplementary material for: Ultrasensitive Photoelectrochemical Immunoassay Strategy Based on Bi2S3/Ag2S for the Detection of the Inflammation Marker Procalcitonin
Source: Biosensors (Basel). 2023 Mar 10;13(3):366. doi: 10.3390/bios13030366 (PMC10046654; doi:10.3390/bios13030366)
Supplement: Supplementary file 1 [file biosensors-13-00366-s001.zip › biosensors-2214480-supplementary.pdf]

Article

Ultrasensitive Photoelectrochemical Immunoassay Strategy Based on Bi_2S_3/Ag_2S for the Detection of the Inflammation Marker Procalcitonin

1. Materials

Thioglycolic acid (TGA) was obtained from Macklin Reagent Co., Ltd. (Shanghai, China). 1-ethyl-3-(3-dimethylaminopropyl) carbodiimide hydrochloride (EDC) and N-hydroxysuccinimide (NHS) were obtained from Aladdin Reagent Database Inc. (Shanghai, China). Bismuth nitrate pentahydrate ($Bi(NO_3)_3{\cdot}5H_2O$), sodium sulfide (Na_2S), silver nitrate ($AgNO_3$), ascorbic acid (AA), absolute ethanol, isopropyl alcohol and acetone were purchased from Sinopharm Chemical Reagent Co., Ltd. (Beijing, China). Phosphate buffered solution (PBS, 1/15 mol·L^{-1} KH_2PO_4 and 1/15 mol·L^{-1} Na_2HPO_4) containing AA was used as an electrolyte for the PEC measurements. All other chemicals in the experiment were analytical grade and were used as received without further purification.

2. Apparatus

Scanning electron microscope (SEM) images and energy dispersive spectroscopy (EDS) were obtained by using a field-emission SEM (Zeiss, Gemini 300, Germany). Electrochemical impedance spectroscopy (EIS) analysis was performed with an RST5200F electrochemical workstation (Zhengzhou Shiruisi Technology Co., Ltd, China) with a three-electrode system in a 5.0 mmol·L^{-1} $[Fe(CN)_6]^{3-/4-}$ solution containing 0.10 mol·L^{-1} KCl. UV-vis diffuse reflectance spectrum measurements were performed with a Shimadzu UV-3101PC spectrometer (Japan). All PEC experiments were measured on a CHI760E electrochemical workstation (Chenhua Instrument Shanghai Co., Ltd, China) by using a conventional three-electrode system comprising of a saturated calomel electrode as reference electrode, a platinum wire as a counter-electrode, and the as-prepared SnO_2/BiOI/Ag_2S modified ITO electrode (2.5×1.0 cm^2) as working electrode.

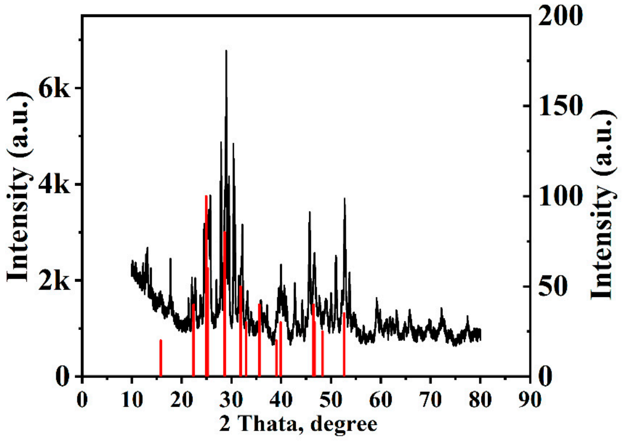

Figure S1. The XRD spectrum of Bi_2S_3.

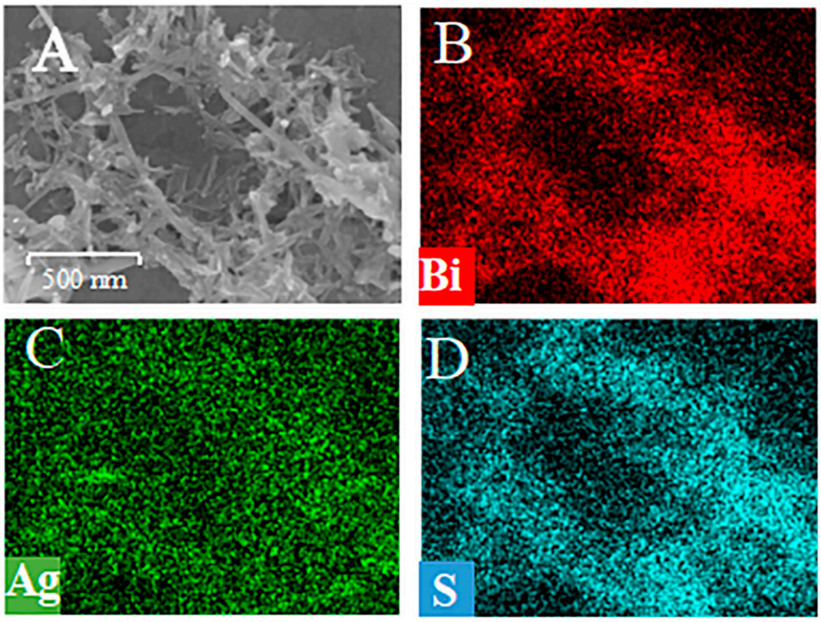

Figure S2. The SEM image of Bi_2S_3/Ag_2S composites (A); the corresponding EDS mapping images of Bi_2S_3/Ag_2S composites with elements of Bi (B), Ag (C), and S (D).

Table S1. Comparison for the performance of the proposed and referenced methods for PCT detection.

Methods	linear range (ng mL^{-1})	detection limit (pg mL^{-1})	References
Electrochemical immunosensor	0.001-100	0.3	1
Fiber optic nanogold-linked immunosorbent assay	0.0001~50	0.083	2
A dual-mode PCT electrochemical immunosensor	0.001~100	0.095	3
Double antibody sandwich method	0.1~10	250	4
SERS magnetic immunoassay	0~20	42	5
Gold-based paper sensor	0.49-13.90	100	6
Signal-Off ECL sensing model	0.0005~50	0.18	This work

Table S2. The results of the PCT determination in human serum samples.

Content in samples (ng·mL^{-1})	Added content (ng·mL^{-1})	Average content (n=11) (ng·mL^{-1})	RSD (n=11, %)	Recovery (%)
0.100	0.100	0.199	3.6	90%
	0.200	0.298	3.1	98%
0.500	0.300	0.804	4.1	101%
	0.600	1.013	3.7	86 %

References

(1) Miao, J.; Du, K.; Li, X.; Xu, X.; Dong, X.; Fang, J.; Cao, W.; Wei, Q. Ratiometric electrochemical immunosensor for the detection of procalcitonin based on the ratios of SiO_2-Fc-COOH–Au and UiO-66-TB complexes. *Biosens. Bioelectron.* **2021**, *171*, 112713.

(2) Li, Y.; Liu, L.; Liu, X.; Ren, Y.; Xu, K.; Zhang, N.; Sun, X.; Yang, X.; Ren, X.; Wei, Q. A dual-mode PCT electrochemical immunosensor with $CuCo_2S_4$ bimetallic sulfides as enhancer,. *Biosens. Bioelectron.* **2020**, *163*, 112280.
(3) Chiang, C.; Huang, T.; Wang, C.; Huang, C.; Tsai, T.; Yu, S.; Chen, Y.; Hong, S.; Hsu, C.; Chang, T. Fiber optic nanogold-linked immunosorbent assay for rapid detection of procalcitonin at femtomolar concentration level. *Biosens. Bioelectron.* **2020**, *151*, 111871.
(4) Zhou, Y.; Shao, X.; Han, Y.; Zhang, H. Detection of procalcitonin (PCT) using the double antibody sandwich method based on fluorescence resonance energy transfer between upconversion nanoparticles and quantum dots. *Anal. Methods* **2018**, *10*, 1015-1022.
(5) Wang, X.; Ma, L.; Hu, C.; Liu, T.; Sun, S.; Liu, X.; Guan, M. Simultaneous quantitative detection of IL-6 and PCT using SERS magnetic immunoassay with sandwich structure. *Nanotechnology* **2021**, *32*, 255702.
(6) Xu, X.; Lei, X.; Ye, L.; Song, S.; Liu, L.; Xu, L.; Xu, C.; Hua, K. Gold-based paper sensor for sensitive detection of procalcitonin in clinical samples. *Chinese J. Anal. Chem.* **2022**, *50*, 100062.
